# Strain Transfer Mechanisms and Mechanical Properties of Optical Fiber Cables

**DOI:** 10.3390/s22249966

**Published:** 2022-12-17

**Authors:** Shenghan Zhang, Han Liu, Sanjay Govindjee, Matthew J. DeJong

**Affiliations:** 1 Department of Civil and Environmental Engineering, Hong Kong University of Science and Technology, Clear Water Bay, Kowloon, Hong Kong; 2 Department of Civil and Environmental Engineering, University of California, Berkeley, CA 94720, USA

**Keywords:** distributed fiber optic sensing, concrete structure, strain transfer mechanism, mechanical properties

## Abstract

Understanding the strain transfer mechanism is required to interpret strain sensing results for fiber optic cables. The strain transfer mechanism for fiber optic cables embedded in cementitious materials has yet to be thoroughly investigated experimentally. Interpretation of fiber optic sensing results is of particular concern when there is a displacement discontinuity. This study investigates the strain transfer mechanism for different types of fiber optic cables while embedded in concrete cubes, sustaining a boundary condition which features a displacement discontinuity. The strain transfer mechanisms for different cables are compared under increasing strain levels. Under cyclic loading, the nonlinear behavior of the force–displacement relation and of the strain distribution in the fiber optic cable are discussed. The mechanical properties of the fiber optic cables are presented and discussed. A parameter is proposed to quantify the strain transfer length. The results of this study will assist researchers and engineers to select appropriate cables for strain sensing and interpret the fiber optic sensing results.

## 1. Introduction

Distributed fiber optic sensing (DFOS) can be used for damage detection in civil infrastructure [1,2,3]. Recent developments in DFOS bring increasingly higher accuracy, finer resolution and longer coverage [4,5], and make it possible to quantify infrastructure behavior at unprecedented resolution. For instance, Optical Frequency Domain Reflectometry (OFDR) offers sub-millimeter level spatial resolution and microstrain level accuracy [6], and has attracted significant attention from the research community [7,8,9,10,11,12,13]. Despite the high resolution and accuracy, interpretation of fiber optic sensing results remains a challenge because DFOS measures the strain in the fiber optic core, which is transferred from the structure through the fiber optic cable jacket and coating (hereafter referred to as the “coating layer” for convenience). In particular, at locations of sharp changes in strain (e.g., displacement discontinuities), the strain transfer mechanism between the structure and the optical fiber needs to be understood in order to interpret DFOS results [14].

The strain transfer mechanism for fiber optic sensors was first studied by Ansari et al. [15], after which numerous researchers have contributed to the topic [16,17,18,19,20,21]. Recently, the advent of OFDR has provided the ability to measure strain at the refined spatial resolution required to obtain the ground truth of strain in the fiber optic core. Based on OFDR, Bassil et al. [22] proposed a multi-layer analytical model and investigated concrete crack sensing with experiments. Zhang et al. [23] developed a mechanical model composed of springs, which compares well with the analytical model and can further consider the nonlinear behaviour of the interface between the fiber optic cable and the structure. The mechanical model can also be used to decompose the DFOS strain distribution under multiple cracks. Liu et al. [14] developed a new deconvolution method which can be used to mathematically decompose and accurately measure crack widths for both sensitive fiber-optic cables and cables with reduced sensitivity but better survivability. Falcetelli et al. [24] investigated the strain transfer mechanism with two fiber optic cables bonded with epoxy to the surface of aluminum specimens. The experimental results compare well with analytical results and finite element simulation. Zheng et al. [25] investigated the strain transfer effect under both single- and bi-linear strain gradients with a fiber optic cable bonded with epoxy to the surface of an aluminum alloy tube, and compared experimental observations with closed form solutions.

Despite the aforementioned efforts and progress, there are still several limitations with existing research: (i) Reinforced concrete sensing often requires embedding fiber optic sensors in concrete. However, there is still a lack of detailed experimental investigations using fiber optic sensors embedded in cementitious materials. Many of the existing studies were based on fiber optic cables bonded to the surface with epoxy. However, surface-bonded sensors feature a different strain transfer mechanism [22]. (ii) Current analytical models compare well with experimental observations in the linear range. However, there is still limited research on the strain transfer mechanism when nonlinear behavior occurs, either in the fiber optic cable or at the interface. (iii) There is scarce information on the mechanical behavior of fiber optic cables. Most research investigates the cable behavior under displacement boundary conditions and neglects the cable-structure interaction. To quantify the error while neglecting the cable’s contribution to the structural behavior, force information of the fiber optic cable is needed. Additionally, to design proper embedment lengths for fiber optic sensors, the interface cohesion and the cable strength are needed. (iv) To obtain strain information from OFDR, a calibration coefficient is required to convert from sensed spectral shift to the strain level. To determine this coefficient, current calibration approaches (e.g., [24,26]) use the applied displacement to calculate the strain level without considering the strain transfer mechanism. However, this assumption will overestimate the strain in the fiber optic sensor. An error in the calibration coefficient will therefore systematically affect the accuracy of OFDR.

To address these challenges, a testing campaign was conducted involving different fiber optic cables subjected to a displacement discontinuity. The objectives are to: (i) investigate the linear and nonlinear strain transfer mechanisms of fiber optic cables embedded in concrete under increasing strain levels and cyclic loading; (ii) propose an index with physical meaning to quantify the smoothing effect of fiber optic cables subjected to displacement discontinuity, which can be used by engineers to estimate the strain transfer length and to select appropriate fiber optic cables for specific applications; (iii) investigate the mechanical behavior of various types of fiber optic cables, including the stiffness, hysteresis behavior under cyclic loading, and force relaxation; (iv) propose a new method of fiber optic cable calibration using integration instead of maximum strain. The study employed the ODiSI 6104 Series Sensing Platform from Luna Innovations. Unless noted otherwise, 0.65 mm is used as the gauge pitch.

## 2. Specimen Preparation and Test Setup

**Figure 1:**
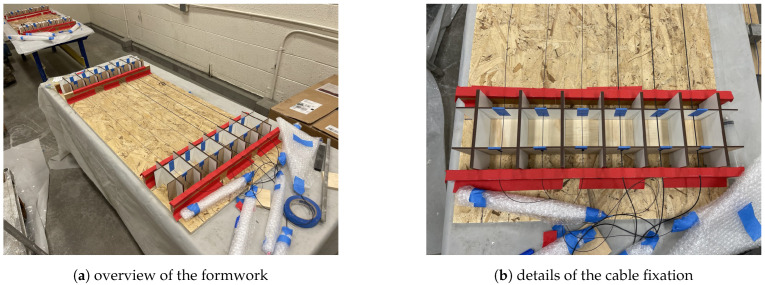
Formwork composed by laser cutting woods.

To create a displacement discontinuity for fiber optic cables, for each fiber optic cable, two concrete cubes (with an edge length of 3 inches, ~76 mm) were cast around two ends of the fiber optic cable, with ~1 m clear spacing between the cubes. The assembled formwork and fiber optic cables are shown before concrete casting in Figure 1. Before fixation of the cable on the formwork, a small amount of pretension is applied to the cable and the tension is maintained by taping the cable to the formwork (Figure 1b) to ensure that the cable is straight during concrete casting and curing (Figure 1).

**Figure 2:**
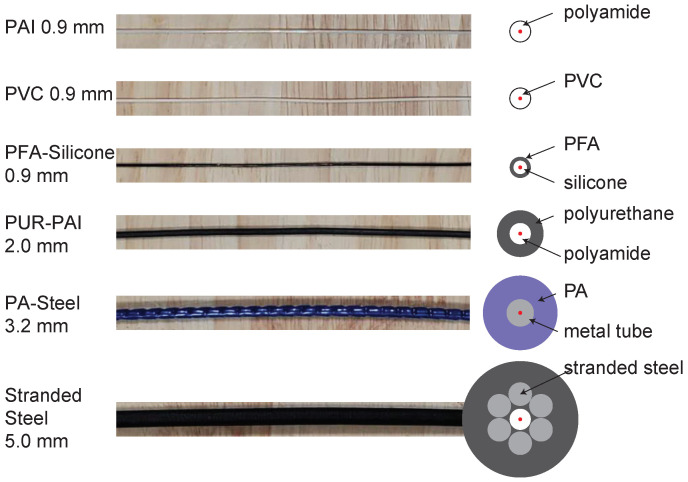
Cables used in the current test campaign [26].

For the concrete, Portland cement (Type IV) was used with a water-to-cement ratio of 0.44. No aggregate was added to minimize the voids during casting. A liquid admixture (${\mathrm{ECLIPSE}}^{®}$) is added to reduce drying shrinkage. In total, three pairs of cubes were cast for each of six different types of cables (i.e., 18 pairs of cubes total). This was conducted in two batches of concrete. Along with the cubes, four 6-inch cylinders were cast for each batch. The six types of fiber optic cables tested in the campaign are PVC 0.9 mm, polyamide (PAI) 0.9 mm, PFA-Silicone 0.9 mm, polyurethane-polyamide (PUR-PAI) 2.0 mm, Stranded Steel 5.0 mm, and PA-Steel 3.2 mm. Please refer to Figure 2 [26] for the structure of different fiber optic cables. The 28-day compressive strength (following ASTM C39) of the cylinders was 40 MPa for the first batch and 39 MPa for the second. The concrete elastic modulus from the cylinder tests was 15 GPa for the first batch and 16 GPa for the second batch.

**Figure 3:**
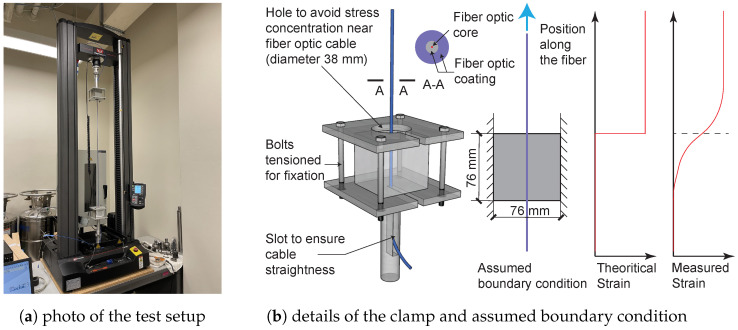
Photos of the calibration rig and details of the clamp.

The test setup (Universal Testing Systems by (${\mathrm{INSTRON}}^{®}$)) with the cable installed is shown in Figure 3a. To connect the concrete cubes to the machine, a pair of clamps were designed and manufactured. The details of the clamp design are shown in Figure 3b.

During the displacement controlled tests, the top cube moves with the load cell while the bottom cube is fixed. Figure 3b also illustrates the theoretical strain in the fiber optic cable (if a perfectly rigid bond is assumed between the fiber and the concrete) along with the real strain considering the smoothing effect of cable coating and potential debonding. Detailed testing protocols and testing results are summarized in the following section. Note that because one pair of cubes (from PA-Steel 3.2 mm cable) was used for loading evaluation and sustained repeated damage, only the results for 17 pairs of cubes are summarized in this study.

## 3. Results and Discussion on Testing Results

### 3.1. Testing Protocol

For each specimen (i.e., pair of cubes), the following four testing protocols were performed selectively.

“Test 1” is a loading-unloading process which allows the examination of the cable behavior under cyclic loading. The top cube was displaced 8 mm, which gives a nominal reference strain of 8000 μϵ for the one meter gauge length. The real strain in the cable varies with the actual length of the sensor, the strain transfer mechanism near the clamp, the interface damage between cable and concrete (if present), and the flexibility of the aluminum clamp (for fiber optic cables with high stiffness, i.e., Stranded Steel 5.0 mm and PA-Steel 3.2 mm). The loading/unloading rate was 0.025 mm/s (nominal reference strain rate of 25 μϵ/s). The unloading was stopped when the force dropped to a small predefined pretension value (i.e., ~5 N for PA-Steel cable 3.2 mm and Stranded Steel 5.0 mm, and ~0.5 N for other cable types). The displacement loading cycle was repeated three times.

“Test 2” investigates the cable behavior (e.g., the residual displacement) under gradually increasing displacement demand. The displacement demand was increased from 1 mm to 8 mm (in 1 mm increments) for each loading cycle. After reaching each target displacement level, the cable was held for 90 s before unloading. The loading/unloading rate was 0.01 mm/s (nominal reference strain rate was 100 μϵ/s).

“Test 3” is a stress relaxation test, which allows for a preliminary estimation of viscous behavior. The cable is loaded up to a pre-specified loading level with three different loading rates, i.e., 0.8 mm/s, 0.2 mm/s and 0.05 mm/s. For the PA-Steel 3.2 mm cable and the Stranded Steel 5.0 mm cable, the pre-specified level was set as 60 N to avoid interface damage; for the other cables, the target level was 8 mm. The displacement was then held at the target displacement/force level for 10 min before unloading.

“Test 4” is a loading-unloading test with increasing displacement demand for determining the cable strength or the interface strength between the cable and concrete, depending on the governing failure mechanism. “Test 4” was conducted on one specimen for each cable type. For the Stranded Steel 5.0 mm cable, “Test 4” is omitted because interface damage was already observed within the 8 mm displacement range in “Test 1” or “Test 2”.

### 3.2. Material and Calibration Parameters

Cable stiffness can be estimated through linear regression from the force F(t) and displacement d(t) from the loading machine at small displacement levels to avoid the influence of nonlinear behavior. Mathematically, estimating cable stiffness *k* can be expressed as a optimization problem

(1) $$\begin{array}{cc} \min_{k,b}\; & \int_{t_{0}}^{t_{N}}\left|F\left(t\right)-k\left[d\left(t\right)-\Delta^{\mathrm{clamp}}F\left(t\right)\right]+b\right|dt\\ s.t.\; & d\left(t_{0}\right)=d_{0},\;d\left(t_{N}\right)=d_{N}\\ \; & k>0 \end{array}$$

in which *k* is the stiffness of the cable, $\left[d_{0},d_{N}\right]$ indicates the displacement range where the stiffness is calculated, taken to be [0.6,0.9] mm for Stranded Steel 5.0 mm and PA-Steel 3.2 mm cable, and [0.2,0.9] mm for all other cables, $\Delta^{\mathrm{clamp}}$ is the flexibility of the clamp, which is only considered when F(t) is large, i.e., for Stranded Steel 5.0 mm and PA-Steel 3.2 mm cable. $\Delta^{\mathrm{clamp}}$ can be obtained by comparing the machine displacement with DIC measurement of the displacement of the end cubes

(2) $$d\left(t\right)-\Delta^{\mathrm{clamp}}F\left(t\right)=d^{\mathrm{DIC}\_\mathrm{up}}\left(t\right)-d^{\mathrm{DIC}\_\mathrm{down}}\left(t\right)$$

in which $d^{\mathrm{DIC}\_\mathrm{up}}\left(t\right)$ is the displacement of the concrete cube on the top measured with DIC (Figure 3a), while $d^{\mathrm{DIC}\_\mathrm{down}}\left(t\right)$ is the displacement of the cube on the bottom measured with DIC. Clamp flexibility is related to the pre-compression applied on the clamp while fastening the bolts. From Equation (2), the clamp flexibility is calculated to be ~$3.2\times 10^{-4}$ mm/N for the Stranded Steel 5.0 mm cable tests, and ~$1.6\times 10^{-4}$ mm/N during the PA-Steel 3.2 mm cable tests.

From the cable stiffness *k*, the elastic modulus can be calculated with

(3) $$E=\frac{kL}{A}$$

in which *A* is the cross section area of the fiber optic cable, *L* is the length of the fiber optic cable, taken as the distance between the inner edges of the clamps. The equivalent elastic modulus of the coating can be estimated by

(4) $$E^{\mathrm{coating}}=\frac{EA-E^{\mathrm{core}}A^{\mathrm{core}}}{A-A^{\mathrm{core}}}$$

in which $A^{\mathrm{core}}$ and $E^{\mathrm{core}}$ are the sectional area and Elastic modulus of the fiber optic core [23].

For OFDR, normally a linear relation is assumed to convert the spectral shift to the measured Fiber Optic (FO) strain $\epsilon^{\mathrm{FO}}$

(5) $$\epsilon^{\mathrm{FO}}=-C_{\epsilon }\Delta \nu$$

in which Δν is the spectral shift (in GHz), obtained with the optical interrogator, and $C_{\epsilon }$ is a calibration coefficient obtained through comparison with the real strain.

**Figure 4:**
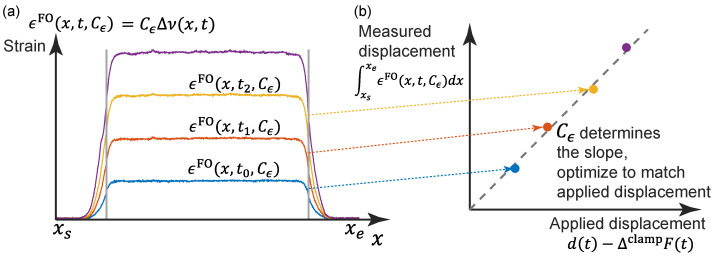
Illustration of the fiber optic cable calibration: (**a**) Strain distributions under different displacement levels (by different colors); (**b**) calibration of $C_{\epsilon }$ by matching the applied displacements.

In previous studies, $C_{\epsilon }$ is calculated by fitting the maximum strain $\epsilon^{\mathrm{FO}}$ with the applied strain, which is estimated by d/L, neglecting the strain transfer mechanism near the clamp. In this study, $C_{\epsilon }$ is determined by matching the measured displacement (by integrating the strain along the whole length of the cable) with the applied displacement (Equation (2)). Mathematically, the calibration coefficient $C_{\epsilon }$ is estimated by solving the following optimization problem (Figure 4)

(6) $$\begin{array}{cc} \min_{C_{\epsilon }}\; & \int_{t_{0}}^{t_{N}}|\left[d\left(t\right)-\Delta^{\mathrm{clamp}}F\left(t\right)\right]-\int_{x_{s}}^{x_{e}}\epsilon^{\mathrm{FO}}\left(x,t,C_{\epsilon }\right)dx|dt\\ s.t.\; & d\left(t_{0}\right)=d_{0},\;d\left(t_{N}\right)=d_{N}\\ \; & C_{\epsilon }>0 \end{array}$$

in which $x_{s}$ and $x_{e}$ refer to the starting/ending point covering the strained length of the sensor, $\left[d_{0},d_{N}\right]$ mm indicates the displacement range within which the calibration coefficient is calculated, chosen to be the same as in Equation (1).

Note that an embedded or surface-bonded clamp is required to use Equation (6). In our previous study [23], a mechanical clamp was used, which squeezes the cable to fix it in place. A large number of invalid data points were observed in this case, which makes integration near the clamp region impossible.

An estimation of the interface strength $c^{\mathrm{interface}}$ or the cable strength $\sigma^{\mathrm{cable}}$ can be obtained from “Test 4”, whichever occurs first

(7) $$c^{\mathrm{interface}}=\frac{F}{2\pi r_{2}h}\;\;\mathrm{or}\;\sigma^{\mathrm{cable}}=\frac{F}{\pi r_{2}^{2}}\;;$$

in which *F* is the maximum force, $r_{2}$ is the radius of the cable, *h* is the height of concrete block (i.e., the embedded length, which is 76.2 mm for current study).

While the subsequent sections present the detailed results and different cable behaviors, Table 1 summarises the elastic modulus of the cable, the strength of the interface or the strength of the cable, and the calibration coefficient (CV represents Coefficient of Variation).

### 3.3. PAI 0.9 mm and PUR-PAI 2.0 mm

**Figure 5:**
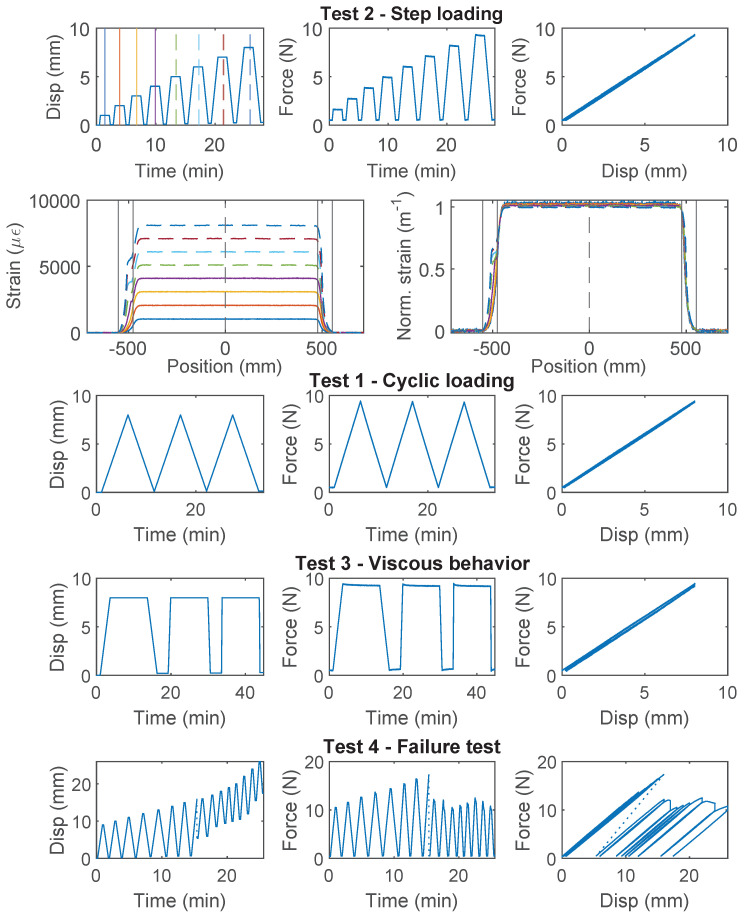
The loading protocol, force–displacement relation and strain distributions under increasing displacement levels (represented by different color lines) for Specimen 3 of the PAI 0.9 mm cable.

**Figure 6:**
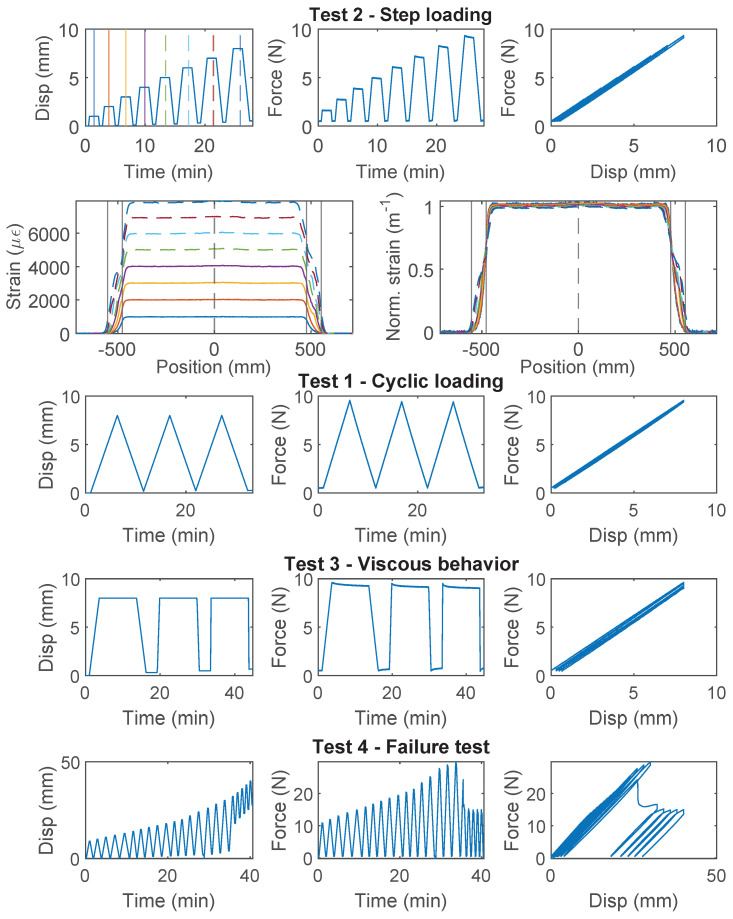
The loading protocol, force–displacement relation and strain distributions under increasing displacement levels (represented by different color lines) for Specimen 3 of the PUR-PAI 2.0 mm cable.

PAI 0.9 mm and PUR-PAI 2.0 mm are designed for strain sensing. Within the 8 mm range of nominal displacement, linear behavior between force and displacement is observed for both cable types (Figure 5 for PAI 0.9 mm, Figure 6 for PUR-PAI 2.0 mm), confirming their suitability for strain sensing under cyclic loading. Note that two more specimens for PAI 0.9 mm and PUR-PAI 2.0 mm were tested under Test 1, Test 2, and Test 3, in varying order. Despite the difference in testing sequence, the force–displacement relations are nearly identical among specimens for each type of cable. Therefore, the force–displacement relations for Specimen 1 and 2 are omitted here.

The strain distributions for the selected specimens of PAI 0.9 mm and PUR-PAI 2.0 mm under increasing displacement demand are presented in Figure 5 and Figure 6, respectively. The strain distributions are taken from the displacement levels indicated by the vertical lines in the upper-left Disp–Time graph, with the corresponding color and line type. To better compare the strain distributions at different displacements, normalized strains are also presented, which are calculated as

(8) $$\epsilon {\left(x\right)}^{\mathrm{Norm}.}=\frac{\epsilon (x)}{\int_{-\infty }^{\infty }\epsilon \left(x_{0}\right)dx_{0}}$$

While the force–displacement relation demonstrates a linear behavior globally, a closer examination of the strain distributions for PUR-PAI 2.0 mm reveals local non-linearity inside the clamp (Figure 6). This local non-linearity can be related with the strain transfer mechanism, which will be discussed in Section 4.2 and Section 4.3.

With “Test 4”, interface damage between the cable and the concrete was observed for both types of cable under increasing displacement demand, i.e., the cables were pulling through the concrete block. For PAI 0.9 mm cable, the initial failure load is 18 N and the residual strength is 12 N, a ~30% decrease. For PUR-PAI 2.0 mm cable, the resistance drops from 28 N to 16 N (~60% decrease). From Equation (7), the residual interface cohesion strength is estimated to be 0.056 MPa for PAI 0.9 mm and 0.059 MPa for PUR-PAI 2.0 mm (Table 1).

### 3.4. PFA-Silicone 0.9 mm

**Figure 7:**
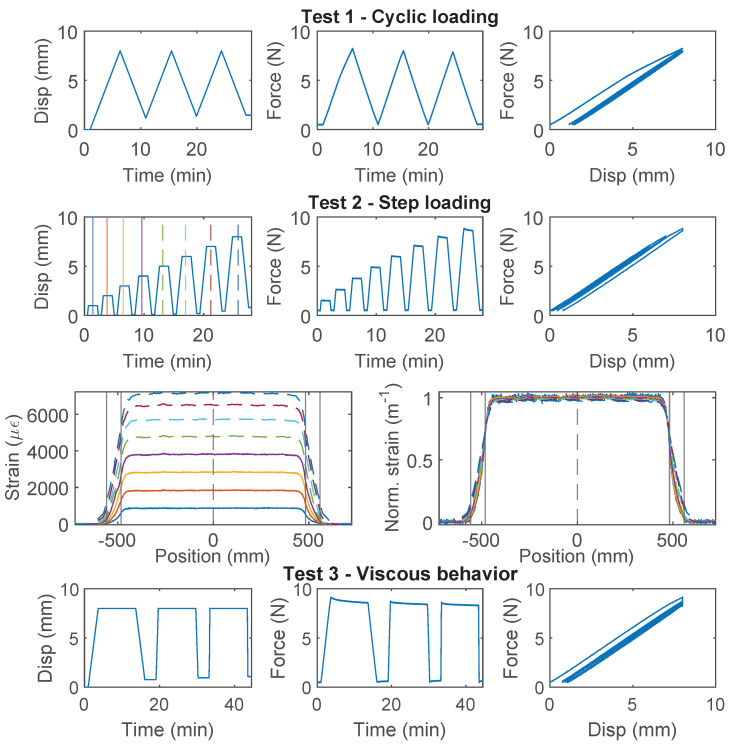
The loading protocol, force–displacement relation, and strain distributions under increasing displacement levels (represented by different color lines) for Specimen 1 of the PFA-Silicone 0.9 mm cable.

The force–displacement relations of PFA-Silicone 0.9 mm demonstrate a higher level of non-linearity. For Specimen 1 (Figure 7), nonlinear behavior is observed during the first loading cycle of “Test 1”, while the second and third loading cycles behave linearly; for “Test 2”, an accumulation of inelastic residual deformation is observed with increasing displacement demand. The force–displacement relation of Specimen 2 was nearly identical to Specimen 1, and therefore omitted here for simplicity. In total, there is ~0.5 mm residual displacement with 8 mm displacement demand.

**Figure 8:**
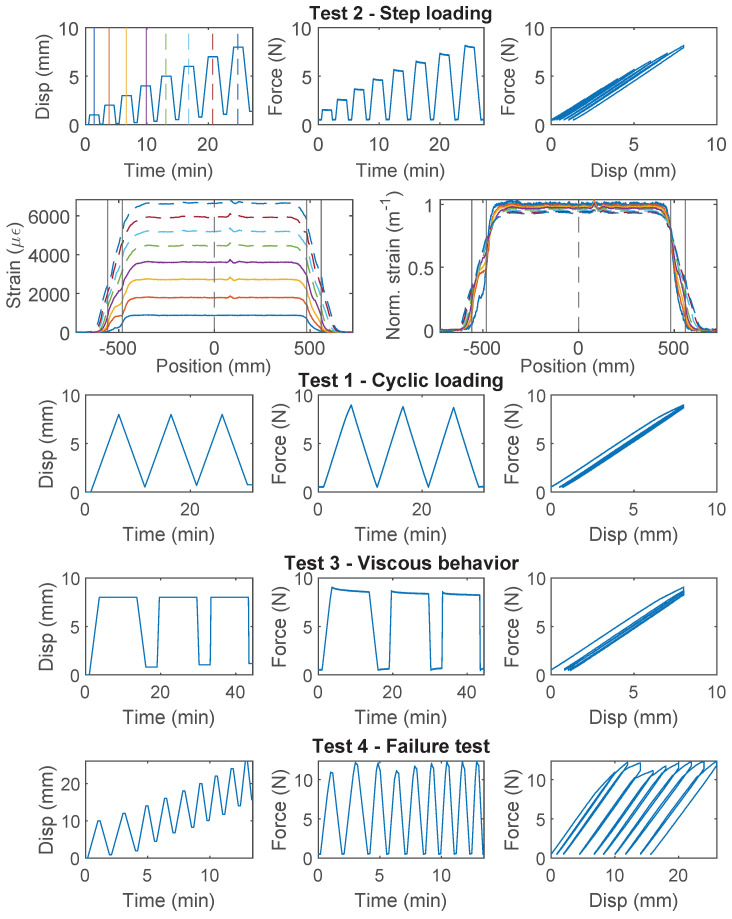
The loading protocol, force–displacement relation and strain distributions under increasing displacement levels (represented by different color lines) for Specimen 3 of the PFA-Silicone 0.9 mm cable.

For Specimen 3 (Figure 8), “Test 2” is performed before “Test 1”. Therefore, a larger residual deformation is observed for Specimen 3 during “Test 2” (~1.0 mm residual displacement as compared to ~0.5 mm for Specimen 1). For “Test 1”, the non-linearity and residual deformation are smaller during the first loading cycle for Specimen 3. The difference demonstrates the influence of testing sequence while using PFA-Silicone 0.9 mm for strain sensing.

The strain distributions at selective displacements are presented in Figure 7 and Figure 8. In contrast to PAI 0.9 mm and PUR-PAI 2.0 mm where the strain distributions are linearly scaling up (Figure 5 and Figure 6), there is a gradual change in the shape of the strain distribution with increasing displacement demand for PFA-Silicone 0.9 mm. This change will be quantified in Section 4.2.

From “Test 4” (Figure 8), the maximum force that can be sustained by the cable is ~12 N, beyond which sliding is observed between the cable and concrete. Different from PAI 0.9 mm and PUR-PAI 2.0 mm, for PFA-Silicone 0.9 mm, the resisting force stays constant after reaching the peak. The estimated cohesion between the cable and concrete is 0.056 MPa.

### 3.5. PVC 0.9 mm

**Figure 9:**
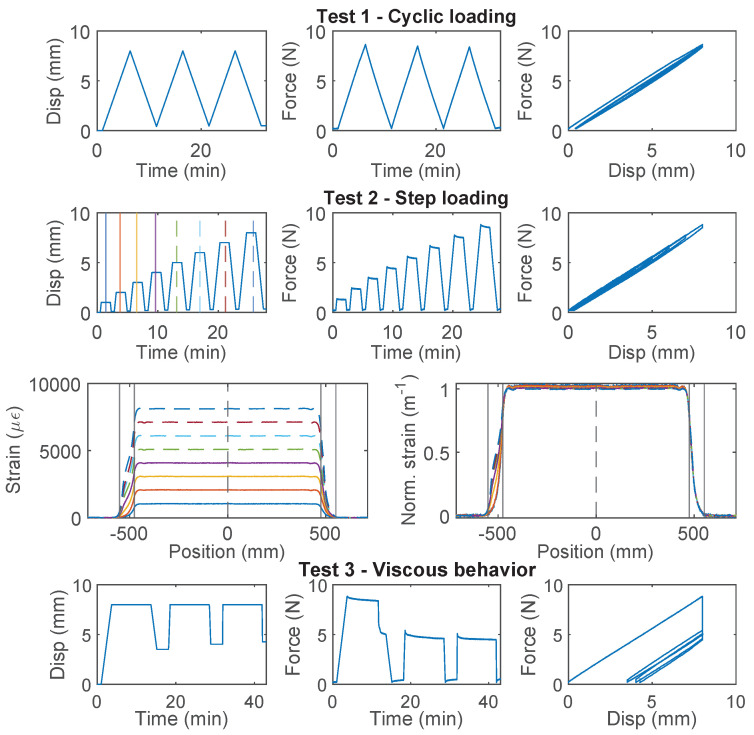
The loading protocol, force–displacement relation and strain distributions under increasing displacement levels (represented by different color lines) for Specimen 1 of the PVC 0.9 mm cable.

**Figure 10:**
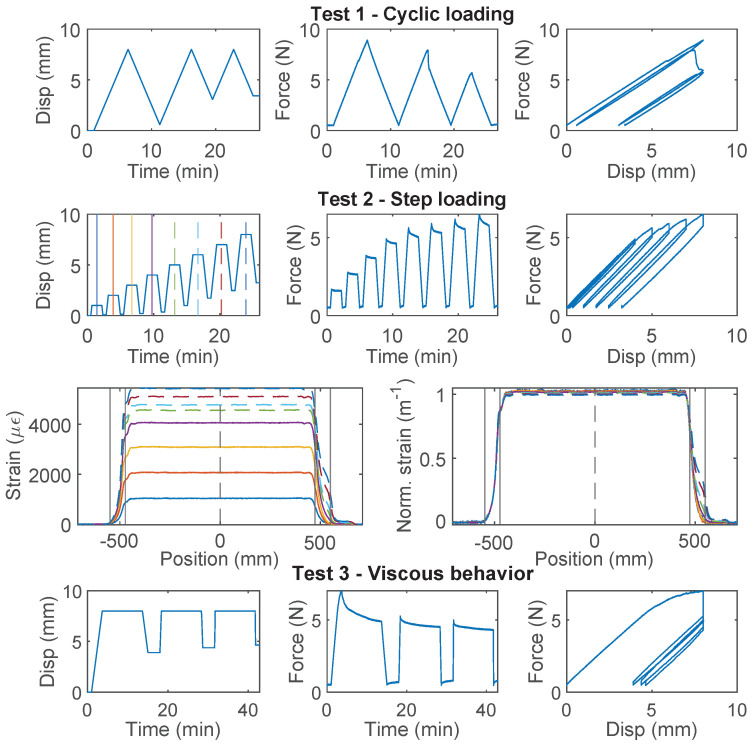
The loading protocol, force–displacement relation and strain distributions under increasing displacement levels (represented by different color lines) for Specimen 2 of the PVC 0.9 mm cable.

**Figure 11:**
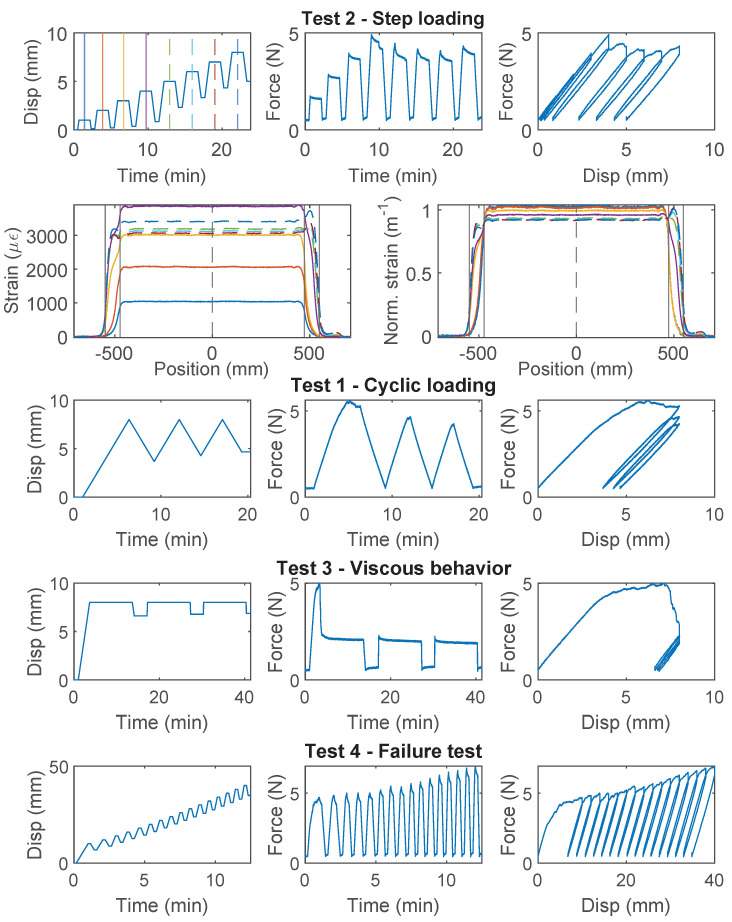
The loading protocol, force–displacement relation and strain distributions under increasing displacement levels (represented by different color lines) for Specimen 3 of the PVC 0.9 mm cable.

For PVC 0.9 mm cable, which is a common cable used for communication and is occasionally used for structural sensing, significant nonlinear behavior is observed for all three specimens (Figure 9, Figure 10 and Figure 11). For Specimen 1, the force–displacement relation was nearly linear during “Test 1” and “Test 2”, while there was a sudden drop of the load while holding displacement during “Test 3”. For specimen 2, the nonlinear deviation of the force–displacement relation was observed during the second loading cycle of “Test 1”, while for Specimen 3, the nonlinear deviation occurred during the first loading cycle of “Test 1”. This difference of cable behavior indicates a large variation of cable properties.

Specimen 2 and Specimen 3 both entered the nonlinear range after “Test 1”, and their force–displacement relations then displayed different characteristics: a gradual increase of force is observed with increasing displacement demand for Specimen 2, while the maximum force stays constant after reaching ~4.5 N for Specimen 3.

The difference between Specimen 2 and 3 during “Test 2” is further revealed by comparing the strain distributions (Figure 10 and Figure 11). For Specimen 2, with the increase of displacement, the maximum strain increases slower compared to Specimen 1. The slower increasing maximum strain is accompanied by stiffness softening in the force–displacement relation (“Test 2” of Specimen 2, Figure 10). Given that the cable structure of PVC 0.9 mm features a very weak interface between the PVC coating and the fiber optic core, this phenomenon is likely caused by the partial slippage between the fiber optic core and the coating layer.

For Specimen 3, the maximum strain is reached at 4 mm displacement, and the strain distribution gradually shifts to the outer edges of the two clamps with increasing displacement demand, indicating complete slippage between the fiber optic core and the coating. Specimen 3 sustains further increasing displacement demand during “Test 4” (Figure 11) and the measured force gradually increases. Significant nonlinear deformation of the coating is observed at the end of “Test 4”.

As a first estimation of cable strength, PVC 0.9 mm cable is estimated to behave linearly within 3800 μϵ (Specimen 3), based on which the cable strength is estimated to be ~6.3 MPa from Equation (7).

### 3.6. Stranded Steel 5.0 mm and PA-Steel 3.2 mm

Stranded Steel 5.0 mm and PA-Steel 3.2 mm have a very stiff coating layer (Figure 2). Due to their robustness, these cables are commonly adopted for on-site monitoring projects [1,27]. Quantifying the strain transferring mechanism of these cables will facilitate data interpretation for onsite projects which feature displacement discontinuity.

**Figure 12:**
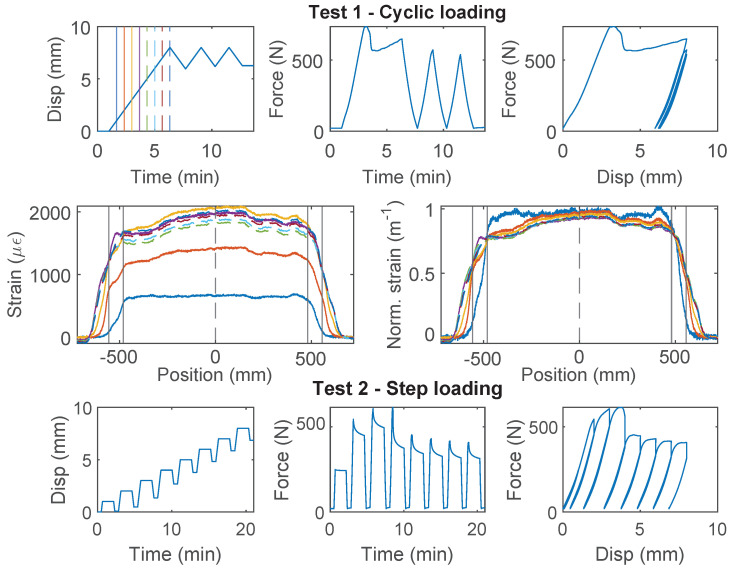
The loading protocol, force–displacement relation and strain distributions under increasing displacement levels (represented by different color lines) for Specimen 1 of the Stranded Steel 5.0 mm cable.

For the Stranded Steel 5.0 mm cable, Specimen 1 starts with “Test 1” (Figure 12), during which interface failure occurs at ~750 N (~3 mm of displacement demand). The force drops to ~600 N after interface damage and slightly increases afterwards. For loading cycle 2 and 3, the same level of displacement demand is applied. Therefore no further damage is observed in “Test 1”. The “strengthening” effect under cycle 1 indicates that the cable interface is not fully broken yet. To test the interface behavior under further displacement demand, “Test 2” applies increasing displacement demands, calculated based on residual displacement from unloading. From the force–displacement relation, the cable interface loses all cohesion at ~600 N (cohesion estimated to 0.5 MPa), and the residual force is ~400 N.

**Figure 13:**
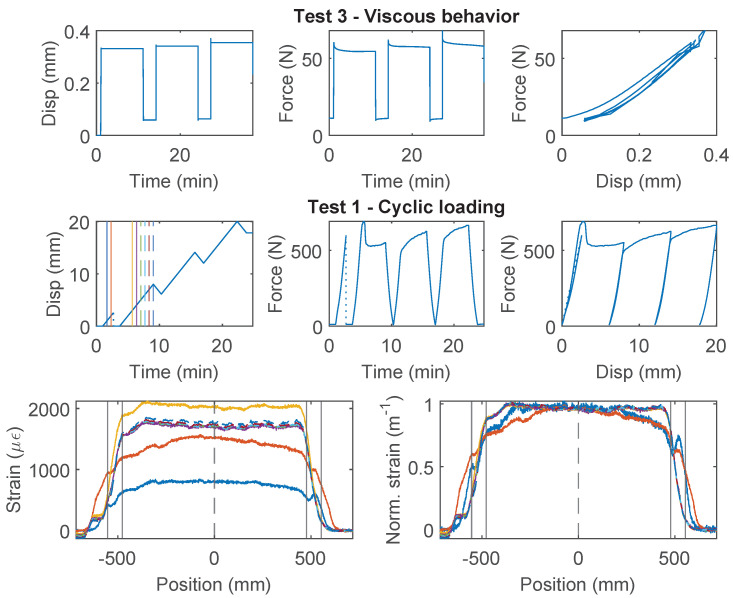
The loading protocol and force–displacement relation for Specimen 2 of the Stranded Steel 5.0 mm cable.

**Figure 14:**
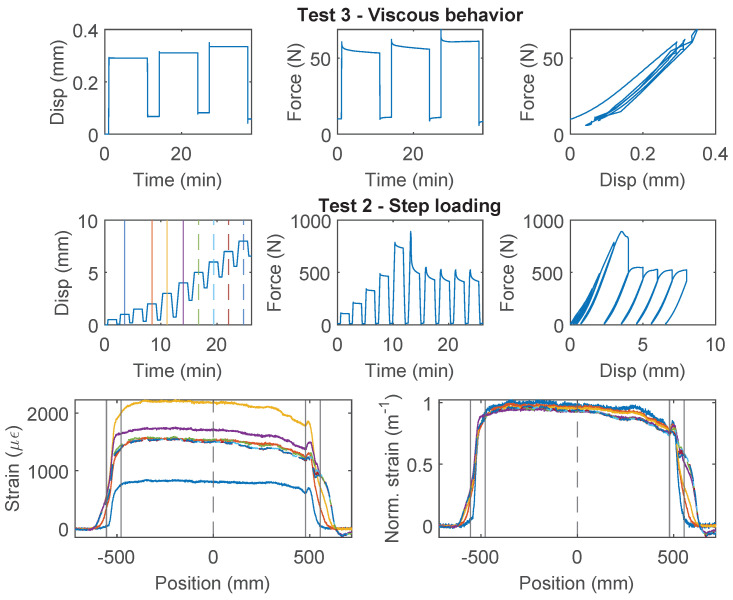
The loading protocol, force–displacement relation and strain distributions under increasing displacement levels (represented by different color lines) for Specimen 3 of the Stranded Steel 5.0 mm cable.

For Specimen 2 and 3 (Figure 13 and Figure 14), “Test 3” is performed first with a target force level of 60 N to prevent interface damage, followed by “Test 1” or “Test 2”, respectively. The viscous behavior from “Test 3” will be discussed with other cables in Section 4.4. Different from Specimen 1, for “Test 1” of Specimen 2, incremental displacement demand is applied, i.e., each loading cycle applies an additional 8 mm of displacement increase from the residual displacement. Note that the test stopped unexpectedly at ~600 N due to a loading machine issue and was restarted afterwards (Figure 13). The behavior of the Specimen 2 is similar to Specimen 1, i.e., the strengthening effect is observed after the initial damage of the interface (drop of the force). However, for Specimen 3, no strengthening effect is observed, i.e., the force stays constant after initial damage.

The difference of the force displacement behaviour can also be revealed by examining the strain distributions at varying displacement demands for the three specimens (Figure 12, Figure 13 and Figure 14). For Specimen 1 and Specimen 2, damage occurs at both clamps at the initial stage, represented by an expanded strain distribution, while the concentration of damage is more clearly observed at the right clamp for Specimen 3.

**Figure 15:**
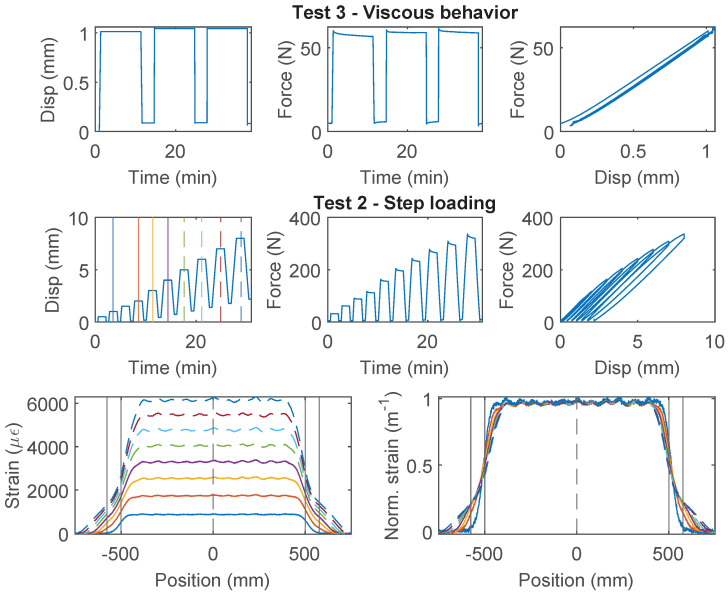
The loading protocol, force–displacement relation and strain distributions under increasing displacement levels (represented by different color lines) for Specimen 3 of the PA-Steel 3.2 mm cable.

**Figure 16:**
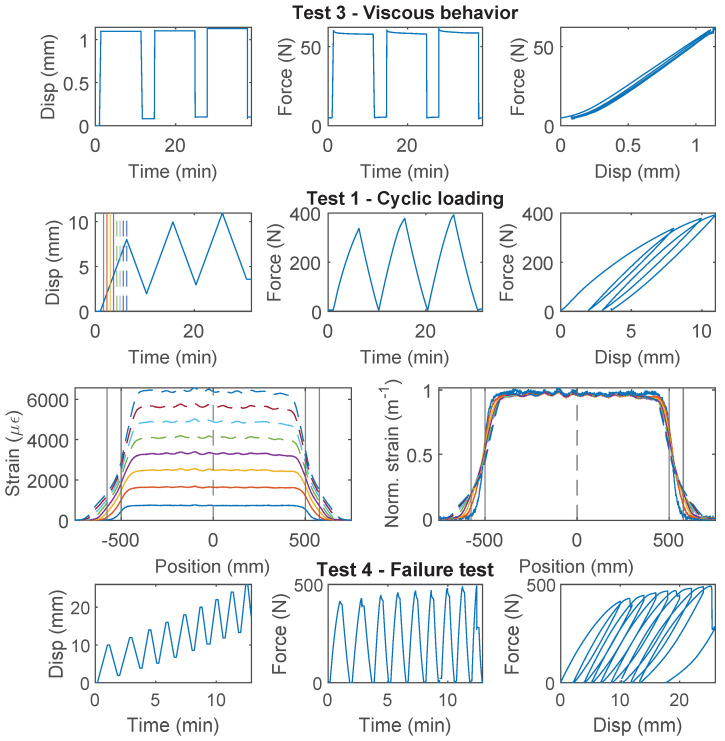
The loading protocol, force–displacement relation and strain distributions under increasing displacement levels (represented by different color lines) for Specimen 2 of the PA-Steel 3.2 mm cable.

For PA-Steel 3.2 mm, only the test results for two specimens (Specimen 2 and 3) are presented in Figure 15 and Figure 16, because Specimen 1 sustained repeated damage during pre-test evaluation. Similar to Specimen 2 of the Stranded Steel 5.0 mm cable, both specimens of PA-Steel 3.2 mm started with “Test 3” with a target force level of 60 N, after which “Test 1” and “Test 4” were performed for Specimen 2, while “Test 2” was conducted for Specimen 3. From “Test 2” of Specimen 3 (Figure 15), a gradual accumulation of residual displacement is observed with increasing displacement demand. Compared with other cables, PA-Steel 3.2 mm shows a unique hysteresis feature. The energy dissipation during the loading and unloading cycle can be attributed to nonlinear outer sheath deformation. The nonlinear behavior of the cable is also manifested in the strain distributions in Figure 15 and Figure 16, in which the strain distribution become “smoother” under increasing displacement demand. A parameter, strain transfer length, will be proposed in Section 4.2 to quantify this smoothing effect.

The PA-Steel 3.2 mm cable is tested until failure with “Test 4” (Specimen 2, Figure 16). Different from Stranded Steel 5.0 mm which presents a interface damage, the PA-Steel 3.2 mm cable itself failed at 480 N, upon which the PA layer at the outer cable sheath broke, while the steel tube and the fiber core remained intact (there is still signal transmitted through the failure point after unloading). The mechanical properties for the Stranded Steel 5.0 mm cable and the PA-Steel 3.2 mm cable are summarized in Table 1.

## 4. Comparison of Different Types of Cable

### 4.1. Linear Distribution and Comparison with Mechanical Model

To explain the smoothing effect (i.e., the relatively smooth strain measurement that occurs at theoretically sharp discontinuities), a mechanical model [23] can be used to simulate the cable behavior. To better represent the structure of the fiber optic cable, we introduce another parameter $\alpha_{\mathrm{out}}$, representing the shear stiffness reduction between the fiber coating layer and the surrounding matrix (concrete here). The shear stiffness for the inner and outer layer of the fiber optic cable are therefore calculated as

(9) $$k^{om}=\alpha_{\mathrm{out}}\frac{G^{\mathrm{eqv}}s\pi \left(r_{2}+r_{12}\right)}{r_{2}-r_{12}}\;\mathrm{and}\;k^{mc}=\alpha_{\mathrm{in}}\frac{G^{\mathrm{eqv}}s\pi \left(r_{12}+r_{1}\right)}{r_{12}-r_{1}}$$

in which $r_{12}=\frac{r_{1}\;+\;r_{2}}{2}$, $r_{1}$ and $r_{2}$ are the radius of the fiber optic core and the radius of the cable, respectively, *s* is the spacing between springs [23], $\alpha_{\mathrm{in}}$ and $\alpha_{\mathrm{out}}$ are the stiffness reduction factors for the two shear spring layers, and $G^{\mathrm{eqv}}$ is the equivalent shear modulus of the coating layer. As in [23], $G^{\mathrm{eqv}}$ is deduced from

(10) $$G^{\mathrm{eqv}}=\frac{E^{\mathrm{coating}}}{2(1+\nu )}$$

in which $E^{\mathrm{coating}}$ is calculated from the stiffness of the cable from the experimental results (Equation (4), Table 2). The poisson ratio ν is assumed to be 0.3 [19,23].

**Figure 17:**
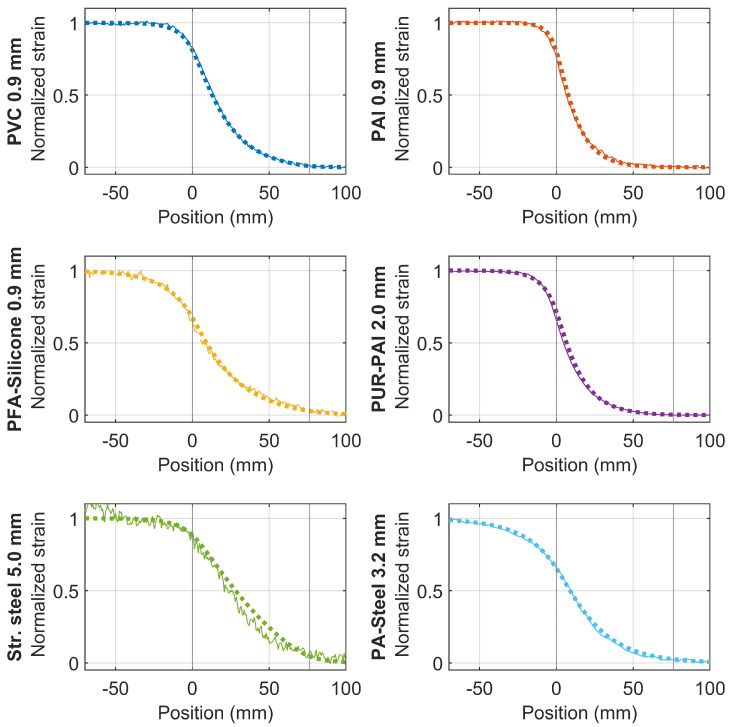
Strain distribution of different cables (solid lines) with comparison to mechanical model (dotted line).

To compare the smoothing effects for different types of cable, Figure 17 summarizes the normalized strain distributions in the elastic stage (when the displacement demand is below 1 mm), and the strain distribution from the mechanical model (“dotted lines”). The location of the clamp is indicated by the two vertical lines in the figure. By adjusting $\alpha_{\mathrm{out}}$ and $\alpha_{\mathrm{in}}$, the mechanical model can simulate the experimental results reasonably well (Figure 17), demonstrating the ability of the mechanical model to reveal the strain smoothing effect in the linear range.

The difference of the smoothing effect for different fiber optic cables is evident in Figure 17. To quantify this effect, we define the strain transfer length with a parameter *l* in the following section.

### 4.2. Strain Transfer Length l

**Figure 18:**
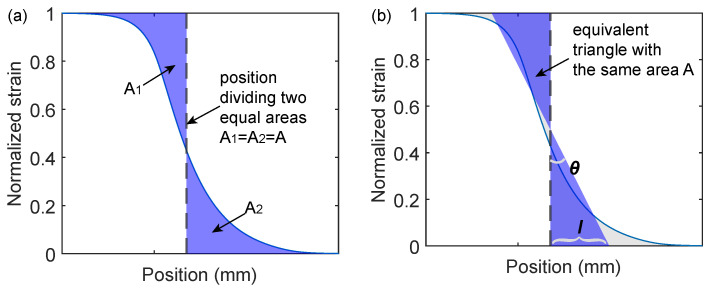
Parameters *l* and θ indicating smoothing effect of the coating layer: (**a**) Illustrative strain distribution; (**b**) equivalent strain distribution and physical meaning of *l* and θ.

Considering a strained fiber optic cable entering a clamp, the strain in the fiber optic cable gradually decreases from a full tension value $\epsilon_{\max}$ to zero. Given a point $x_{0}$ within the transition zone, two areas $A_{1}$ and $A_{2}$ are defined as (Figure 18a)

(11) $$A_{1}\left(x_{0}\right)=\int_{x_{\mathrm{start}}}^{x_{0}}\left(\epsilon_{\max}-\epsilon \left(x\right)\right)dx\;\mathrm{and}\;A_{2}\left(x_{0}\right)=\int_{x_{0}}^{x_{\mathrm{end}}}\left(\epsilon \left(x\right)\right)dx$$

in which $x_{\mathrm{start}}$ is chosen as a point satisfying $\epsilon \left(x_{\mathrm{start}}\right)=\epsilon_{\max}$, and $x_{\mathrm{end}}$ is a point with no strain, i.e., $\epsilon (x_{\mathrm{end}})=0$. $x_{\mathrm{mid}}$ is defined as the $x_{0}$ equally dividing $A_{1}$ and $A_{2}$

(12) $$A_{1}\left(x_{\mathrm{mid}}\right)=A_{2}\left(x_{\mathrm{mid}}\right)\equiv A.$$

Note that *A* correlates with the smoothing effect; A=0 refers to the situation when there is sharp change of strain (theoretical strain in Figure 3b). Based on *A*, the strain transfer length *l* defined as

(13) $$l=\frac{2A}{\epsilon_{\max}/2}=\frac{4A}{\epsilon_{\max}}\;\left[\mathrm{mm}\right].$$

Note that *l* has units of length and is normalized with (and therefore independent of) the strain level in the fiber optic cable. To explore the physical meaning of *l*, an equivalent triangle is defined with the same area *A* (Figure 18b). *l* is the length of the triangle base which gives the length of the strain distribution.

**Figure 19:**
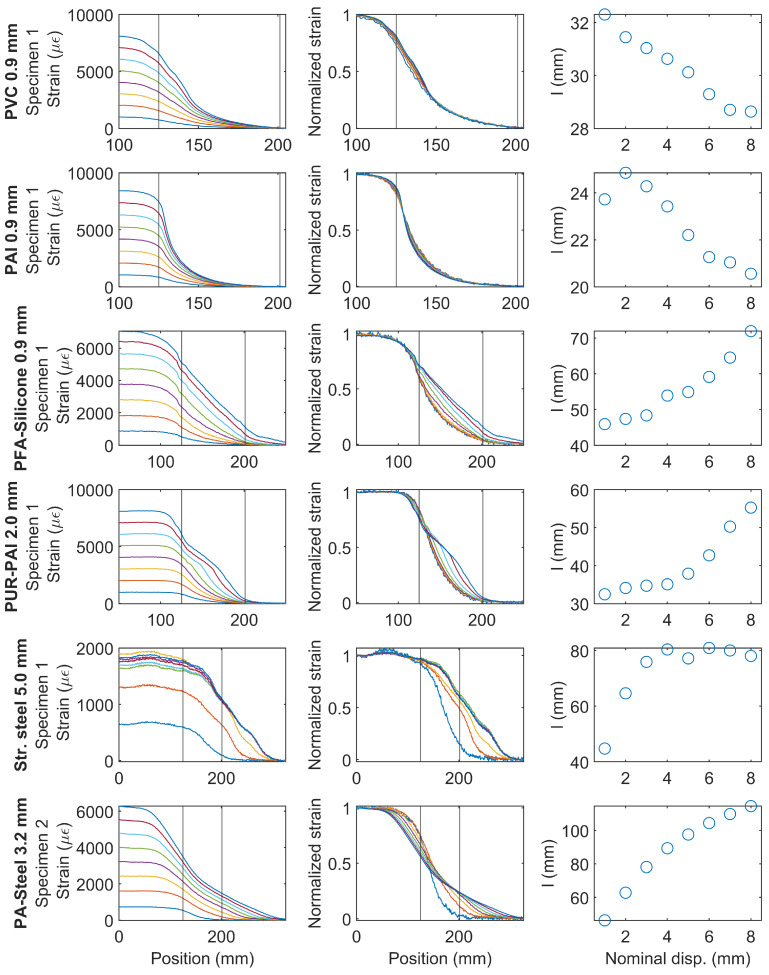
Sample strain distributions for each type of cable and smoothing parameters *l* (different color lines represent strain distributions under increasing displacement levels).

Figure 19 summarizes the strain variation for different cable types. The first column of Figure 19 presents the strain distribution under increasing nominal displacement demand and the second column shows the normalized strain distribution (normalized by maximum strain). The two vertical lines in the first and the second column indicate the position of the clamp. The third column presents the change of *l* with the nominal displacement.

For PVC 0.9 mm and for PAI 0.9 mm, the normalized strains are similar for the different displacement levels; *l* gradually decreases (a relatively small amount) indicating the normalized strain distribution is slightly narrower at higher strain demand.

For PFA-Silicone 0.9 mm and for PUR-PAI 2.0 mm, the shape of strain distributions gradually changes with increasing nominal displacement. Up to a displacement of 3 mm, within which the cable behavior is linear, *l* stays roughly constant, with only a slight increase. With larger displacement demand, the nonlinear cable response results in a significant increase in *l*.

For Stranded Steel 5.0 mm, *l* increases from 30 mm to 80 mm (~170%) with nominal displacement increasing from 1 mm to 3 mm. Because of interface damage, the strain level in the fiber optic cable stays approximately constant with further increase of displacement, therefore *l* also stabilizes with increasing nominal displacement.

For PA-Steel 3.2 mm, nonlinear behavior starts after a displacement of 1 mm. As a result, with the gradual increase of nominal displacement, *l* increases from 40 mm to 120 mm (~200% increase), indicating a much larger strain transfer length at high strain demand.

**Figure 20:**
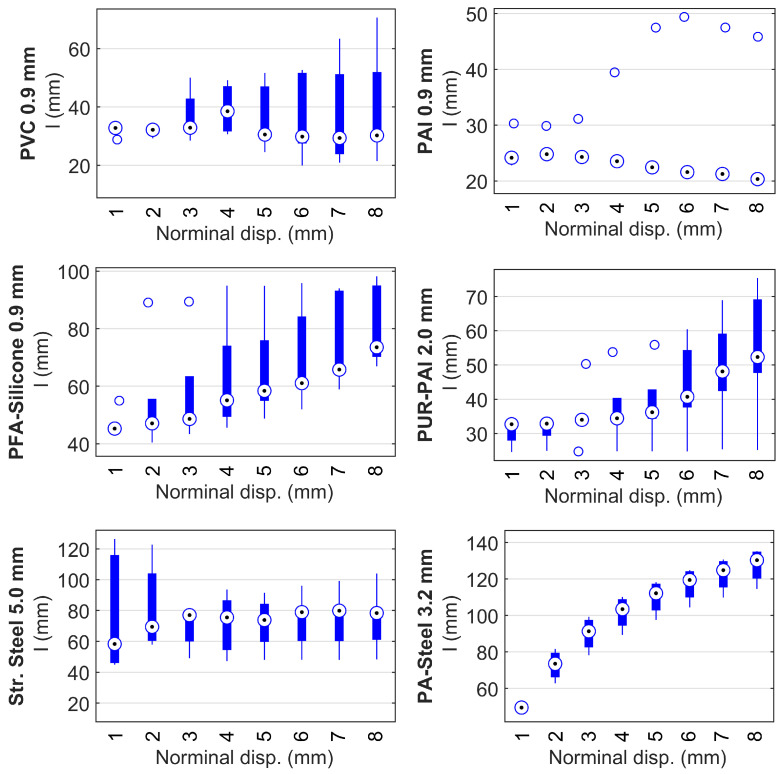
Box plot for strain transfer length *l*.

Figure 19 presents the results of one cable around one clamp per cable type. To evaluate the statistical reliability for the smoothing parameter *l*, Figure 20 shows the strain transfer length *l* calculated from both sides of the clamp and from all specimens. The conclusions are similar: for PAI 0.9 mm, *l* is relatively constant although there is a slight decrease of *l* indicating that the normalized strain distribution is narrower with increasing displacement; for PVC 0.9 mm, the median of *l* is also relatively constant with regard to the displacement level, while a much larger variation is observed; for PFA-Silicone 0.9 mm, PUR-PAI 2.0 mm, and PA-Steel 3.2 mm, *l* significantly increases with displacement demand, indicating a smoother strain distribution with increasing strain level; for Stranded Steel 5.0 mm, *l* increases with displacement before the displacement reaches 2 mm, after which *l* stabilizes because of the interface damage.

Following Figure 20, the statistical values for different cables are summarized in Table 3. Note that the “Initial *l*” column represents the response at a displacement of 1 mm, and best represents the strain transfer length during linear behavior, while the “Final *l*” column represents the strain transfer length when the applied displacement reaches 8 mm. *l* under higher displacement demand is different because the strain distribution is influenced by both the nonlinear behavior of the cable (depending on the cable type) and the interface damage between cable and concrete.

**Figure 21:**
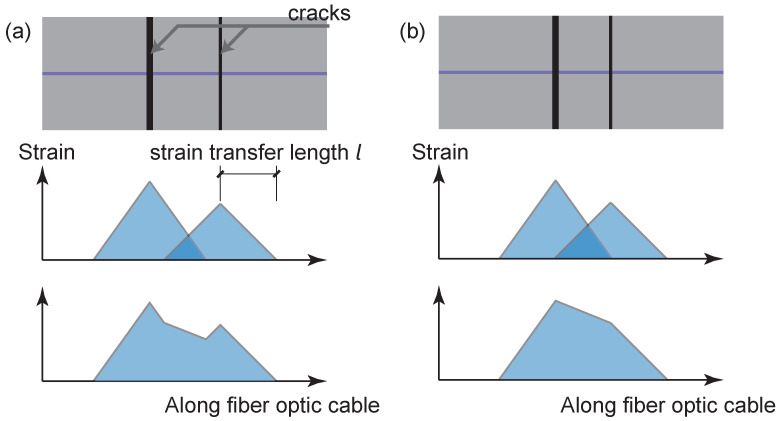
Illustration of the strain superposition in the fiber optic cable: (**a**) enough spacing to distinguish different cracks; (**b**) insufficient spacing between cracks.

For crack quantification, the transfer length can be used as an indicator to estimate the crack spacing at which different cracks can be distinguished. This is illustrated by Figure 21.

Note that the above illustration is a rough estimation based on three assumptions: (1) the strain distribution follows a triangular shape under the cracking scenario; (2) length of the strain distribution on one side of the crack is equal to the strain transfer length l; (3) the law of superposition is satisfied, which assumes that there is no damage to the interface between the cable and the concrete.

To quantify the crack widths of multiple cracks, the mechanical model proposed in [23] can be used, by minimizing the difference between measured strain distribution and simulated strain distribution. However, establishing the mechanical model requires estimating the mechanical properties of the fiber optic cable [23]. Figure 17 demonstrates that the mechanical model (with parameters estimated by this study) compares well with the actual measurements.

### 4.3. Shear Stress Distribution along the Fiber Optic Core

The above discussion demonstrates the effectiveness of *l* in quantifying the overall transfer length (smoothing effect) of the cable in the presence of the displacement discontinuity. To evaluate the local behavior for different cables within the strain transfer length, the shear stress τ(x) around the fiber optic core is calculated as

(14) $$\tau \left(x\right)=\frac{E^{\mathrm{core}}\pi r_{1}^{2}}{2\pi r_{1}}\frac{d\epsilon (x)}{dx}=\frac{E^{\mathrm{core}}r_{1}}{2}\frac{d\epsilon (x)}{dx}$$

in which $E^{\mathrm{core}}=70$ GPa and $r_{1}=0.0625$ mm are the elastic modulus and radius of fiber optic core, ϵ(x) is the strain along the fiber optic core. τ(x) can be normalized based on the total shear force obtained from integration

(15) $$\tau^{\mathrm{normalized}}\left(x\right)=\frac{\tau (x)}{\int \tau \left(x_{0}\right)2\pi rdx_{0}}$$

**Figure 22:**
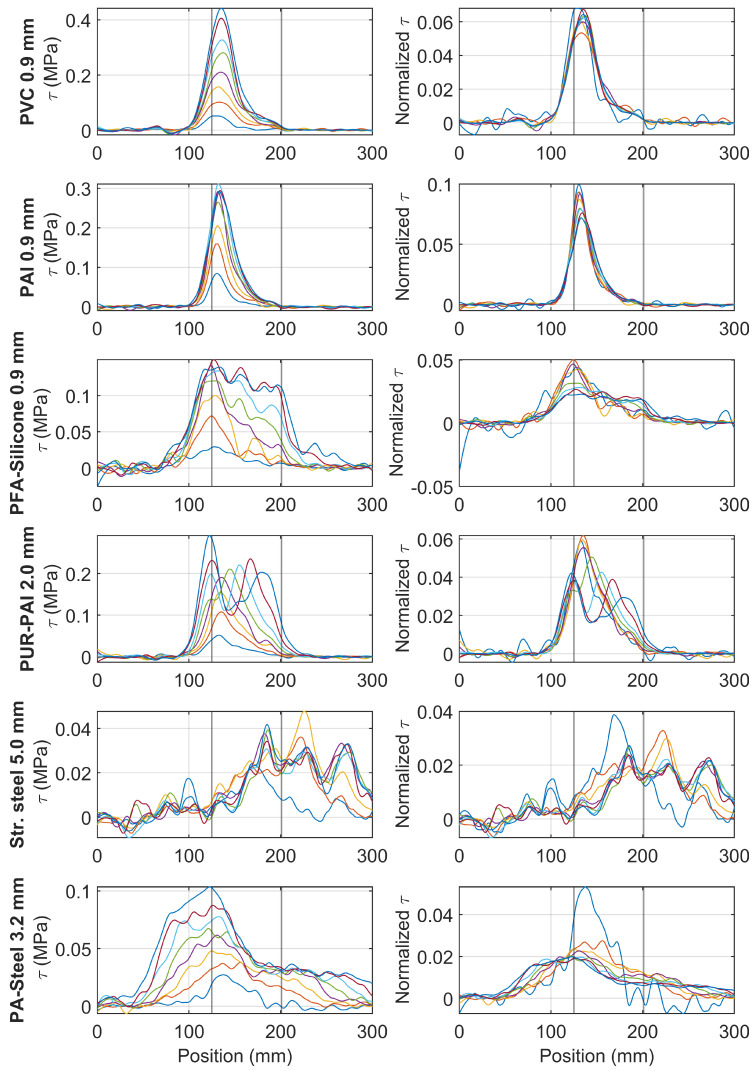
Shear stress distribution along the fiber optic core with increasing displacement demand (different color lines represent strain distributions under increasing displacement levels).

From ϵ(x) in Figure 19, Figure 22 summarizes the shear stress τ(x) and $\tau^{\mathrm{normalized}}\left(x\right)$ for different cable types under varying displacement demand. It can be observed that: (i) for PVC 0.9 mm and PAI 0.9 mm, for which *l* remains approximately constant with increasing displacement, the shape of the shear stress distribution also remains unchanged; (ii) for PFA-Silicon 0.9 mm, with increasing displacement demand, the shear stress gradually propagates through the clamp region and also distributes more uniformly; (iii) for PUR-PAI 2.0 mm, double peaks are observed with increasing displacement demand, which likely indicates a changing failure mechanism; (iv) for PA-Steel 3.2 mm, the shear stress distribution is shifted inside the clamp with increasing displacement demand, which can be caused by the steel tube yielding within the cube coating layers.

### 4.4. Discussion of Visco-Elastic Behavior

**Figure 23:**
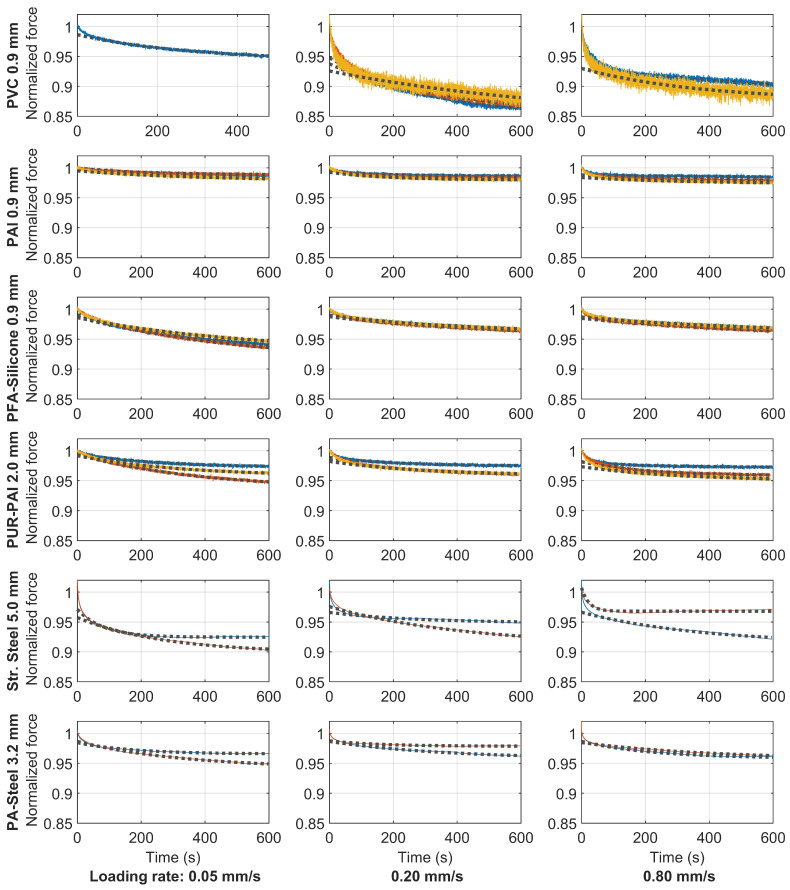
Comparison of force relaxation while holding displacement for different types of cable (color lines represent measurements of different samples while dotted lines represent the exponential fit to the measurements).

In “Test 3” after reaching the specified loading level, the peak displacements were held constant to enable investigation of visco-elastic effects for each specimen. Figure 23 shows the relaxation curves for all of the cables; the three columns represent different loading rates. For the Stranded Steel 5.0 mm and PA-Steel 3.2 mm, only two relaxation tests are conducted for each loading rate. For the other cables, three force relaxation tests are conducted for each loading rate. However, due to cable slippage and other abnormal behaviors (e.g., Specimen 3 of cable PVC 0.9 mm in Figure 11), certain tests are eliminated from Figure 23. For the ease of comparison, the maximum force is normalized to be “1” before force relaxation. The measurement noise is relatively large for the cable PVC 0.9 mm, PAI 0.9 mm, PFA-Silicone 0.9 mm, PUR-PAI 2.0 mm because of relative low force values for these cables.

Because of the high noise and the large variation observed for the same type of cable, e.g., the two force relaxation curves deviate significantly for PA-Steel 3.2 mm under the same loading rate, this paper only gives a first estimation of the relaxation time by fitting an exponential function to the force relaxation curve, using

(16) $$\begin{array}{cc} \min_{a,b,c}\; & \int_{t_{s}}^{t_{e}}\left|F\left(t\right)-\left(a+be^{-ct}\right)\right|dt\\ s.t.\; & a,b,c>0 \end{array}$$

in which F(t) is the measured force history from experiments, $a+be^{-ct}$ is the exponential function fitting the experimental curve, and *a*, *b* are *c* are variables to be optimized. From the theory of linear viscosity, it can be easily deduced that the relaxation time $\tau =\frac{1}{c}$ and, when the relaxation time is infinite, the estimated force is *a*. $t_{s}$ and $t_{e}$ were determined through a trail-and-error process in the range of 120 s to 600 s. The fitted exponential curves are plotted by dotted lines in Figure 23. The relaxation time, the force drop in 10 min (experimental observation) and the expected force drop under long term relaxation (estimated from *a*) are summarized in Table 4.

The current findings give a useful indication of the relative viscosity of the various cables as well as the order of magnitude of potential creep effects when using fiber optic cables for long-term monitoring applications. However, note that this study only presents a first evaluation of the viscous behavior. In reality, the viscosity comes from two sources, i.e., shear stress relaxation and normal stress relaxation. More detailed research is required to distinguish viscosity from different sources and to develop a nonlinear model for the viscous behavior of the fiber optic cable.

## 5. Conclusions

The goal of the current study was to examine the strain transfer mechanism for various fiber optic cables that were subjected to varying strain demands. The test campaign reproduced a discontinuous displacement boundary condition for fiber optic cables embedded in concrete. Under various loading protocols, six different types of fiber optic cables with various cable structures and strain transfer mechanisms were studied. The key findings are:

- Under the tested strain levels (up to ~8000 μϵ nominal strain), the force–displacement relations varied significantly for the considered cables. For PAI 0.9 mm and PUR-PAI 2.0 mm, the results indicated linear behavior. For PVC 0.9 mm cables, large variations and nonlinear behavior was observed at early stages of testing (~3000 μϵ). For PFA-Silicone 0.9 mm, PA-Steel 3.2 mm, and Stranded Steel 5.0 mm cable, varying levels of residual strain are observed under cyclic loading.
- With the increase of displacement demand, fiber optic cables sustained either interface damage or cable failure. Under the current embedding condition (~76 mm of embedding length with ~40 MPa concrete strength), interface damage between the cable and concrete is observed for PFA-Silicone 0.9 mm, PUR-PAI 2.0 mm, PFA-Silicone 0.9 mm and Stranded Steel 5.0 mm. The interface cohesion for Stranded Steel 5.0 mm is ~0.5 MPa, while the cohesion for the other other three cables is estimated to be ~0.05 MPa. For PA-Steel 3.2 mm, cable failure is observed with the estimation of cable strength to be ~60 MPa.
- The strain transfer length *l* is proposed to quantify the smoothing effect of different fiber optic cables under displacement discontinuity. For PAI 0.9 mm, *l* was the shortest and stayed constant at ~25 mm. For PVC 0.9 mm, the median value of *l* stays constant at ~35 mm while the variation increases with displacement. For PFA-Silicone 0.9 mm, PUR-PAI 2.0 mm, and PA-Steel 3.2 mm, *l* started between 30–50 mm and then significantly increased at higher strain levels due to nonlinear behavior. For Stranded Steel 5.0 mm, the median of *l* increases from 60 to 80 mm when the nominal displacement reaches 3 mm, after which *l* remains constant due to interface damage. In general, *l* provides a useful quantification of the strain transfer length that should be expected when interpreting fiber optic strain measurements.
- A more accurate method for cable calibration, i.e., calibrating the coefficient transforming the measured spectral shift to a strain level, is proposed. The new calibration method considers the influence of the strain transfer region near the cable fixations through integrating the strain along the whole cable length, instead of relying on a single strain value. Calibration coefficients are provided for different types of fiber optic cable.
- A modified mechanical model was able to reproduce the linear strain transfer mechanism of all six fiber optic cables. This model was used to infer the mechanical properties of the different cable coatings, which ranged from 70 MPa to 10 GPa. From the experiments, the relaxation time and viscosity for different fiber optic cables were also evaluated. For all cables other than PVC 0.9 mm, the total force drop (from viscous effects) is estimated to be less than 10%.

These results provide a basis for both the selection of fiber optic sensing cables and the interpretation of fiber optic sensing results, particularly for projects involving abrupt changes in displacement or strain. 

## Figures and Tables

**Table 1:** Mechanical parameters and calibration coefficient.

Cable Type	Elastic Modulus		Strength			Calibration Coefficient		
	Mean (GPa)	CV	Cohesion (MPa)	Cable (N)	Nominal Failure Strain	$C_{\epsilon }$ (μϵ/GHz)	Scale Factor	CV
PVC 0.9 mm	1.7	0.021	>0.019	4	0.0038	6.74	1.01	0.006
PAI 0.9 mm	1.7	0.004	0.056	>12	>0.017	6.66	1.00	0.001
PFA-Silicone 0.9 mm	1.6	0.020	0.056	>12	>0.012	5.79	0.87	0.022
PUR-PAI 2.0 mm	0.35	0.003	0.059	>28	>0.031	6.57	0.99	0.015
Stranded Steel 5.0 mm	10.8	0.15	0.50	>600	>0.0036	6.28	0.94	0.103
PA-Steel 3.2 mm	7.6	–	>0.63	480	0.024	6.48	0.97	–

**Table 2:** Parameters used in the mechanical model.

Cable Type	Mechanical Model		
	$E^{\mathrm{coating}}$ (MPa)	$\alpha_{\mathrm{in}}$	$\alpha_{\mathrm{out}}$
PVC 0.9 mm	391	0.0020	0.0012
PAI 0.9 mm	348	0.0050	0.0030
PFA-Silicone 0.9 mm	241	0.0005	0.0048
PUR-PAI 2.0 mm	73	0.0120	0.0350
Stranded Steel 5.0 mm	10,769	0.0005	0.0025
PA-Steel 3.2 mm	7486	0.0002	0.0200

**Table 3:** Strain transfer length *l* for different types of cable.

Cable Type	Initial *l* (mm)		Final *l* (mm)	
	Mean	CV	Mean	CV
PVC 0.9 mm	30	0.08	37	0.52
PAI 0.9 mm	26	0.12	24	0.42
PFA-Silicone 0.9 mm	47	0.04	79	0.15
PUR-PAI 2.0 mm	29	0.13	54	0.33
Stranded Steel 5.0 mm	78	0.59	80	0.37
PA-Steel 3.2 mm	47	0.06	129	0.06

**Table 4:** Relaxation time and force drop for different types of cable.

Cable Type	Relaxation Time (s)		Force Drop in 10 min		Final Force Drop (Estimated)	
	Mean	CV	Mean	CV	Mean	CV
PVC 0.9 mm	536	0.61	11%	0.30	13%	0.29
PAI 0.9 mm	331	0.68	2%	0.29	2%	0.25
PFA-Silicone 0.9 mm	557	0.29	4%	0.31	6%	0.29
PUR-PAI 2.0 mm	273	0.24	4%	0.22	4%	0.31
Stranded Steel 5.0 mm	241	0.66	7%	0.36	7%	0.36
PA-Steel 3.2 mm	278	0.38	4%	0.26	4%	0.30

## Data Availability

The data presented in this study are openly available in https://doi.org/10.6078/D1JH8F.
